# "*Candidatus Rickettsia kellyi*," India

**DOI:** 10.3201/eid1203.050853

**Published:** 2006-03

**Authors:** Jean-Marc Rolain, Elizabeth Mathai, Hubert Lepidi, Hosaagrahara R. Somashekar, Leni G. Mathew, John A.J. Prakash, Didier Raoult

**Affiliations:** * Université de la Méditerranée, Marseille, France;; † Christian Medical College and Hospital, Tamil Nadu, India

**Keywords:** Rickettsia, human rickettsiosis, India, skin biopsy, children, dispatch

## Abstract

We report the first laboratory-confirmed human infection due to a new rickettsial genotype in India, "*Candidatus Rickettsia kellyi*," in a 1-year-old boy with fever and maculopapular rash. The diagnosis was made by serologic testing, polymerase chain reaction detection, and immunohistochemical testing of the organism from a skin biopsy specimen.

Human rickettsioses are infections of emerging importance in India, where increasing numbers of cases among residents and travelers have been reported recently (*1**,**2*). Nevertheless, these diseases are not well described in the literature and, to date, only serologic evidence of rickettsial infections has been reported, including murine typhus, scrub typhus, and unidentified spotted fever group (SFG) rickettsiosis (*1**,**3**,**4*). Moreover, the results of serologic testing are presumptive and should be interpreted with caution. SFG rickettsiosis is seldom diagnosed in India, probably because of a low index of suspicion and a relative lack of diagnostic facilities. Specific diagnostic methods are needed to identify unexpected SFG agents either by polymerase chain reaction (PCR) or by culture (*5*).

Few reports of rickettsioses in children from southern India have been reported. Here we report the case of a 1-year-old boy with a new SFG rickettsiosis characterized by a maculopapular rash on the palms and soles. The diagnosis was confirmed by serologic testing, molecular detection, and immunohistochemical testing of a skin biopsy specimen. We propose the name "*Candidatus Rickettsia kellyi*" in honor of Professor Patrick Kelly, who has greatly contributed to the current knowledge of rickettsiae throughout the world.

## The Study

A 1-year-old boy from Thiruppathur, Tamil Nadu, India, was brought for treatment; he had exhibited fever for 10 days and a maculopapular rash on the face and chest that had spread rapidly to the trunk and limbs. The rash was also on his palms and soles. No tick bite was noted. A skin biopsy was taken from a maculopapular lesion. Laboratory tests showed a leukocyte count of 15,300/mm^3^, hemoglobin level of 9.2 g/dL, and normal platelet count, and normal cerebrospinal fluid was seen by lumbar puncture. The patient was given doxycycline syrup and cefotaxime because the diagnosis was not definitive and the boy was very ill. The boy responded dramatically and eventually recovered. Results of conventional culture of cerebrospinal fluid, skin biopsy specimens, and blood culture were negative.

**Figure 1:**
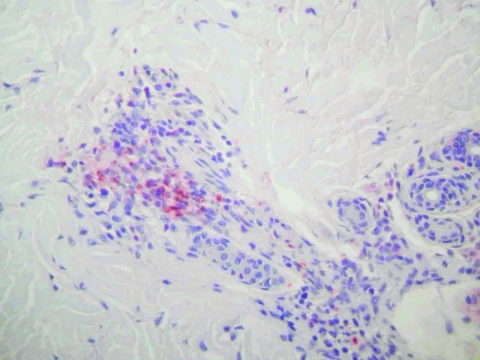
Immunohistochemical results showing rickettsiae in inflammatory infiltrates of the dermis (polyclonal rabbit anti-*Rickettsia* sp. antibody using a dilution of 1:1,000 and hematoxylin counterstain; original magnification ×250).

With Weil-Felix agglutination assay, the serum sample taken at admission was weakly positive with OX-2 antigen (titer 40) and negative for OXK and OX-19 antigens, giving presumptive evidence of a rickettsial infection. DNA was extracted from the skin biopsy specimen and used as a template in 2 previously described PCR assays that targeted a portion of the rickettsial *ompA* gene as well as a portion of the rickettsial *gltA* gene, *ompB* gene, and *sca4* genes (*6**,**7*). Amplification products of the expected size were obtained from this extract but not from any concurrently processed control materials. The most closely related rickettsial species was found to be *R. honeï* with pairwise nucleotide sequence homologies of 92.3% for *ompA*, 99.2% for *gltA*, 94.6% for *ompB*, and 99.1% for *sca4*. Histopathologic testing of the skin biopsy specimen showed a leukoclastic vasculitis, and immunohistochemical testing by using a rabbit polyclonal antibody directed against SFG rickettsiae showed positive result (Figure 1).

For specific microimmunofluorescence assay, a panel of 13 rickettsial antigens, including SFG rickettsiae (*R. conorii* subsp. *indica*, *R. japonica*, *R. honei*, *R. helvetica*, *R. slovaca*, AT1 *Rickettsia*, *R. felis*, "*R. heilongjiangensis*"), typhus group rickettsiae (*R. typhi*), *Orientia tsutsugamushi* (strains Gilliam, Kato, Karp, and Kawazaki), *Anaplasma phagocytophilum*, *Ehrlichia chaffeensis*, and *Coxiella burnetii*, was used as previously described (*8**,**9*). The serum sample from the patient at the acute stage of illness was weakly positive for all SFG rickettsia with a negative immunoglobulin G (IgG) titer and a IgM titer from 64 to 128 according to the species tested. Serum was also positive by Western blot analysis as previously reported (*8*), but the species remained undetermined.

## Conclusions

**Figure 2:**
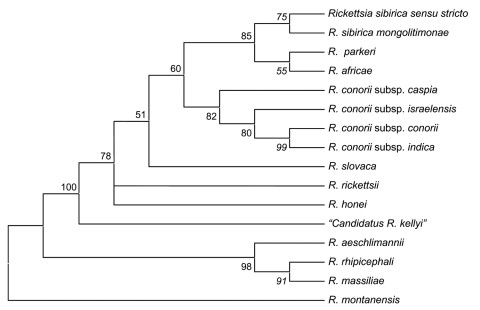
Phylogenetic tree of rickettsiae, including "*Candidatus Rickettsia kellyi*," obtained by comparison of partial sequences of *ompA* with the parsimony method.

Clinical and laboratory data for this patient suggest that he had an SFG rickettsial infection that was confirmed by 3 different testing methods: serologic, immunohistochemical, and molecular based. Although no tick bite and no eschar were noted, the infection could have been acquired from any of a wide variety of arthropods in this area. In India, serologic evidence of human SFG rickettsioses has been found (*1**,**3**,**10*), but the epidemiology of etiologic agents is deduced only by serologic testing performed by using known rickettsial antigens. Our case, to the best of our knowledge, is the first human SFG rickettsiosis case diagnosed in India that was laboratory confirmed by using specific and direct detection of a rickettsial strain. Moreover, according to genetic guidelines for the classification of rickettsial isolates (*6*), our rickettsial strain found in the skin biopsy specimen belongs to a new species. Unfortunately, because the skin biopsy specimen was stored in alcohol, culture and complete phenotypic description of this isolate were not possible. The most closely-related rickettsial strain, according to genetic guidelines, was *R. honeï* as shown in the phylogenetic tree (Figure 2). *R. honeï* is the etiologic agent of Flinders Island (Australia) spotted fever, which was isolated from the blood of 2 patients in 1993 (*11**,**12*). The main reservoir of *R. honeï* was later determined to be *Aponomma hydrosauri*, a reptile tick (*13*). The pathogenicity of the original isolate of *R. honeï* (Thai tick typhus strain TT-118) for humans has not yet been confirmed, but it is possibly responsible for SFG human rickettsiosis in Thailand (*14*). Thus, genotyping of these strains is needed to better understand the epidemiology of SFG rickettsiosis in Asia. Further studies are needed to isolate and establish this new pathogenic SFG rickettsial strain from humans to confirm our case report. Moreover, tick species prevalent in this area of South Asia should be tested to find the rickettsial reservoir and increase understanding of the epidemiology of this rickettsial infection. New pathogens remain to be discovered in India, and new rickettsial diseases represent a challenge.

The partial *ompA* gene sequence of "*Candidatus Rickettsia kellyi*" has been deposited in the GenBank data library under accession no. DQ080005. GenBank accession nos. were TTU59726 for *gltA*, AF123724 for *ompB*,and AF163004 for *sca4*.
